# Increasing Awareness of Hypertensive Patients About Their Blood Pressure Readings During Clinic Visits in a Tertiary Hospital in Riyadh

**DOI:** 10.7759/cureus.33257

**Published:** 2023-01-02

**Authors:** Basim A Almatouq, Abdullah A Alaryni, Abdullah Alghamdi, Qasem A Alhammad, Abdulmalk A Almadhi, Fahad Bin Abbas, Abdullah Z Alnamshan, Ahmed M Khalaf, Atheer M Alyami, Ali Aljafar, Abdullah A Alyousef, Ghaida M Alahmadi

**Affiliations:** 1 Nephrology, King Fahad Medical City, Riyadh, SAU; 2 Internal Medicine, Imam Mohammad Ibn Saud Islamic University, Riyadh, SAU; 3 Internal Medicine, King Fahad Medical City, Riyadh, SAU; 4 Medicine, Imam Mohammad Ibn Saud Islamic University, Riyadh, SAU; 5 Internal Medicine, King Saud Medical City, Riyadh, SAU; 6 Internal Medicine, Dr Sulaiman Al Habib Hospital, Riyadh, SAU

**Keywords:** blood pressure, clinic visits, hypertensive patients, awareness, blood pressure reading, hypertension

## Abstract

Introduction

Hypertension (HTN) is one of the most important cardiovascular risk factors. It is associated with significant complications, such as coronary artery disease, stroke, and chronic kidney disease. Awareness among hypertensive patients regarding their blood pressure (BP) is low in the Kingdom of Saudi Arabia.

Aim

This study aimed to evaluate the awareness of patients regarding their BP readings and to identify which aspects of HTN they needed to be informed about.

Patients and methods

A descriptive cross-sectional study was conducted among hypertensive patients attending outpatient clinics at King Fahad Medical City, Riyadh, Saudi Arabia. An electronic questionnaire was used by a trained physician to collect data from patients during telephone interviews. The information included socio-demographic data (i.e., age, gender, and education), family history, compliance with medications, and BP measurements. Patients were asked to answer questions to assess their awareness of their BP readings.

Results

Of the 475 hypertensive patients included in this study, 32.7% were aged between 56 and 65 years and 60.4% were female. The proportion of patients who had knowledge of their target BP (systolic: 120-129 mmHg; diastolic: 80-84 mmHg) was 74.4%. The significant independent predictors of increased knowledge about BP were a high level of education, regular measurement of BP, and having received education about a healthy lifestyle. The significant independent predictor of decreased knowledge about BP was having an acceptable or poor understanding of chronic BP.

Conclusion

Hypertensive patients visiting the outpatient clinic at King Fahad Hospital had a good understanding of their target BP readings. Educated patients who regularly measured their BP and who received education about a healthy lifestyle tended to exhibit a higher motivation to achieve their BP targets. More research is needed to gain more insights into the knowledge of hypertensive patients and into how they manage their BP to determine the factors that influence their knowledge.

## Introduction

Hypertension (HTN) is one of the most important cardiovascular risk factors that is associated with significant complications such as coronary artery disease, stroke, and renal failure [1]. Awareness among hypertensive patients about their blood pressure (BP) is still low in the Kingdom of Saudi Arabia. The probable reasons for lower awareness may be lower literacy, an unsuitable perception of medical advice, irregular sources of health-related information, or inadequate counseling regarding HTN. In one of the studies, approximately 23% of the patients in this rural population were unaware of the presence of HTN and received no antihypertensive therapy, while 48.0% were not using any antihypertensive medications at the time of this study [2]. Therefore, a comprehensive strategy for reducing hypertensive morbidity should include prevention strategies like increased awareness, early detection, adequate treatment, and strict control of BP among patients in our countries, particularly regarding the risks associated with uncontrolled BP.

Considering these important interventions, we propose evaluating the awareness of HTN among the patients and how the counseling provided by the treating physicians helped identify which area of HTN they needed to learn more about.

## Materials and methods

A descriptive cross-sectional study was conducted on patients attending outpatient clinics at King Fahad Medical City, Riyadh, Saudi Arabia, during the study period of six months. A total of 475 patients were chosen to collect data on their HTN knowledge and awareness, as well as their BP measurement results during a visit. We designed questionnaires for face-to-face data collection, but as some patients cannot attend the clinic due to COVID-19 restrictions, we collected their data during telephone interviews. Verbal consent was taken from each patient before starting each interview, and those who refused the interview were excluded from our study. Both face-to-face and telephone interview data were collected by trained physicians. The information included demographics, family history, compliance with medication, and BP measurements. Patients were asked to answer questions to assess their awareness of their BP readings.

Statistical analysis

Categorical variables were calculated to present numbers and percentages (%). Analysis of the relationship between the knowledge about BP reading among the socio-demographic and other related characteristics of hypertensive patients was conducted using the chi-square test. The generated significant results were then gathered into a multivariate regression model to determine the significant independent predictors associated with knowledge about BP with corresponding odds ratios and a 95% confidence interval (CI). A p-value of 0.05 at 95% CI was used to indicate statistical significance. The data were analyzed using Statistical Packages for Social Sciences (SPSS) version 26 (IBM Corp., Armonk, NY).

## Results

We received survey responses from 465 hypertensive patients. Table 1 presents the socio-demographic characteristics of the patients; 32.7% of the patients were aged between 56 and 65 years old, and 60.4% were female. Patients who held a university degree comprised 33.8% of the cohort. The prevalence of patients who had been diagnosed with chronic HTN was 94.6%; of these patients, almost two-thirds (65.6%) suffered from HTN for five years or more. The proportion of patients who had a family history of chronic HTN was 72.5%. In addition, 47.1% of patients regularly measured their BP.

**Table 1:** Socio-demographic and other related characteristics of the hypertensive patients (n = 465)

Study data	N (%)
Age group	
18-35 years	33 (07.1%)
36-55 years	151 (32.5%)
56-65 years	152 (32.7%)
>65 years	129 (27.7%)
Gender	
Male	184 (39.6%)
Female	281 (60.4%)
Educational level	
Uneducated	81 (17.4%)
Primary school	57 (12.3%)
Middle school	51 (11.0%)
High school	113 (24.3%)
University	157 (33.8%)
Postgraduate	06 (01.3%)
Have you been diagnosed with chronic hypertension?	
Yes	440 (94.6%)
No	11 (02.4%)
I don’t know	14 (03.0%)
Time since diagnosis	
≥5 years	305 (65.6%)
<5 years	160 (34.4%)
Family history of chronic hypertension	
Yes	337 (72.5%)
No	128 (27.5%)
Do you measure your blood pressure regularly?	
Yes	219 (47.1%)
No	246 (52.9%)

Regarding complications and compliance with anti-hypertensive medications, Table 2 shows almost all patients (97.8%) received medications for HTN treatment, and 79.1% of patients took all prescribed medications regularly. Of the patients who were not committed to their treatment plan, the most common (14.8%) reason was forgetfulness. The most common complications of chronic BP were kidney disease (11.6%) and cardiovascular disease (11.6%). Furthermore, 50.3% of the patients indicated regular visits to the emergency department due to HTN.

**Table 2:** Complications and patients’ compliance to hypertension medications (n = 465)

Variables	N (%)
Have you been prescribed medication to treat your blood pressure?	
Yes	455 (97.8%)
No	8 (1.7%)
I do not remember	2 (0.40%)
Do you take all blood pressure medications regularly?	
Yes	368 (79.1%)
No	86 (18.5%)
I do not remember	11 (2.4%)
If you are not committed to your hypertension treatments, what is the reason?	
I forget to take the medicine	69 (14.8%)
I only take it when I feel the symptoms of high blood pressure	58 (12.5%)
I don't want to take the medicine	28 (6.0%)
I don't like it because of the side effects	23 (4.9%)
I want alternative medicine	16 (3.4%)
Other	128 (27.5%)
Committed to treatment	143 (30.8%)
Have you had complications of chronic high blood pressure?	
No	261 (56.1%)
Kidney disease	54 (11.6%)
Cardiovascular disease or coronary heart disease	54 (11.6%)
I don't know	37 (8.0%)
Stroke	27 (5.8%)
Retinal defect	21 (4.5%)
Other complications	11 (2.4%)
Have you visited the emergency department because of high blood pressure?	
Yes	234 (50.3%)
No	231 (49.7%)

**Figure 1:**
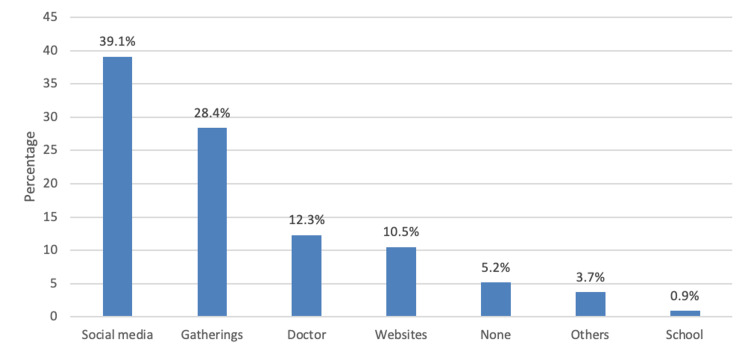
Sources of information on chronic hypertension

As shown in Figure 1, the most common source of chronic HTN information was social media (39.1%) followed by gatherings (28.4%) and doctors (12.3%).

As shown in Table 3, 42.6% of patients demonstrated a good understanding of chronic HTN, 74.4% knew their target BP readings, and 61.1% received education about the goal of their BP readings. In addition, our results revealed that 72.9% of patients received education about healthy lifestyles; however, only 54.2% of patients complied with the recommendations. The most common topic that the patients wanted to know more about was healthy foods and sports (43.4%) and complications of high BP (27.7%). The patients’ preferred learning resource for HTN education was face-to-face discussion (50.5%). More than one-half (54%) of the patients did not know their BP reading; 19.8% of patients knew that their reading was good but did not know the exact numbers.

**Table 3:** Awareness of chronic hypertension and blood pressure readings among hypertensive patients (n = 465)

Awareness statement	N (%)
How would you rate your understanding of your chronic high blood pressure?	
Good	198 (42.6%)
Acceptable	163 (35.1%)
Weak	104 (22.4%)
Do you know the target blood pressure readings?	
Yes	346 (74.4%)
No	119 (25.6%)
Has the goal of blood pressure measurement been discussed with you previously?	
Yes	284 (61.1%)
Probably	181 (38.9%)
Have healthy lifestyles been discussed with you?	
Yes	339 (72.9%)
No	126 (27.1%)
Did you comply with any of the healthy lifestyle modifications?	
Yes	252 (54.2%)
No	168 (36.1%)
I don’t know	45 (9.7%)
What do you want to know more about chronic hypertension?	
Healthy food and sports	202 (43.4%)
Complications of high blood pressure	129 (27.7%)
High blood pressure treatments	103 (22.2%)
All of the above	16 (3.4%)
Nothing	15 (3.2%)
What is your preferred learning resource for hypertension education?	
Written publications	78 (16.8%)
Face-to-face discussions	235 (50.5%)
Explanatory videos	149 (32.0%)
Others	3 (0.60%)
What is the patient's blood pressure reading today?	
Does not know the exact reading	251 (54.0%)
Knows the exact reading	68 (14.6%)
Good but does not know the exact reading	92 (19.8%)
It is not good but does not know the exact reading	22 (4.7%)
Normal reading	24 (5.2%)
Elevated	8 (1.7%)

When evaluating the relationship between the knowledge about BP readings and the socio-demographic characteristics of the patients, we found that the proportion of patients who knew their BP readings was significantly higher among patients who were in compliance with their BP medications (p = 0.001), patients with complications due to HTN (p = 0.036), patients who had perceived good understanding about chronic BP (p < 0.001), and patients who had discussed healthy lifestyles (p < 0.001) (Table 4). In contrast, the proportion of patients who did not know their BP readings was significantly higher among the older age group (p < 0.001), patients who were less educated (p < 0.001), patients who had been diagnosed with HTN for five years or more (p = 0.008), patients who did not regularly measure their BP (p < 0.001), and patients who had never visited the emergency department due to HTN (p = 0.006).

**Table 4:** Relationship between hypertensive patients’ knowledge about target blood pressure and their socio-demographic and other related characteristics (n = 465) ^§^ P-value calculated using chi-square test. ** Significant at p < 0.05.

Factor	Knowledge about blood pressure reading		P-value^§^
	Yes, N (%) ^(n = 346)^	No, N (%) ^(n = 119)^	
Age group			
≤55 years	155 (44.8%)	29 (24.4%)	<0.001**
>55 years	191 (55.2%)	90 (75.6%)	
Gender			
Male	139 (40.2%)	45 (37.8%)	0.650
Female	207 (59.8%)	74 (62.2%)	
Educational level			
High school or lower	200 (57.8%)	102 (85.7%)	<0.001**
University degree or higher	146 (42.2%)	17 (14.3%)	
Time since diagnosis			
≥5 years	215 (62.1%)	90 (75.6%)	0.008**
<5 years	131 (37.9%)	29 (24.4%)	
Family history of chronic hypertension			
Yes	256 (74.0%)	81 (68.1%)	0.212
No	90 (26.0%)	38 (31.9%)	
Regularly measured blood pressure			
Yes	194 (56.1%)	25 (21.0%)	<0.001**
No	152 (43.9%)	94 (79.0%)	
Compliance with blood pressure medication			
Yes	287 (82.9%)	81 (68.1%)	0.001**
No	59 (17.1%)	38 (31.9%)	
Complications due to hypertension			
Yes	142 (41.0%)	62 (52.1%)	0.036**
No	204 (59.0%)	57 (47.9%)	
Visited the emergency department due to high blood pressure			
Yes	187 (54.0%)	47 (39.5%)	0.006**
No	159 (46.0%)	72 (60.5%)	
Rate your understanding of your chronic high blood pressure			
Good	180 (52.0%)	18 (15.1%)	<0.001**
Acceptable	118 (34.1%)	45 (37.8%)	
Weak	48 (13.9%)	56 (47.1%)	
Has a healthy lifestyle been discussed with you?			
Yes	281 (81.2%)	58 (48.7%)	<0.001**
No	65 (18.8%)	61 (51.3%)	

We performed multivariate regression analysis to predict the influence of having knowledge about BP readings (Table 5). Based on the results, we found that having a university degree or higher, regularly measuring BP, and discussing a healthy lifestyle were independent factors that were significantly associated with increased knowledge about BP readings. In contrast, an acceptable or poor understanding of chronic BP was the independent factor significantly associated with decreased knowledge about BP readings. Compared with patients with a high school degree or lower, patients who had a university degree or higher had at least a three-fold increased likelihood of knowing their BP readings (adjusted odds ratio (AOR) = 3.027; 95% CI = 1.569-5.839; p = 0.001). Patients who regularly measured their BP had a 2.9-fold increased likelihood of knowing their BP reading than patients who did not regularly measure their BP (AOR = 2.913; 95% CI = 1.652-5.136; p < 0.001). Similarly, patients who had received education about healthy lifestyles were 3.4 times more likely to know their BP reading compared with patients who did not receive any education about healthy lifestyles (AOR = 3.368; 95% CI = 1.999-5.674; p < 0.001). In contrast, patients who had an adequate understanding of chronic HTN were predicted to have poor knowledge of their BP readings by at least 80% compared with patients with a good level of understanding of chronic HTN (AOR = 0.177; 95% CI = 0.087-0.362; p < 0.001). Knowledge of BP readings was predicted to decrease by at least 50% among those with a poor understanding of chronic HTN (AOR = 0.475; 95% CI = 0.265-0.852; p = 0.013). Other variables, such as age group, time since diagnosis, compliance with BP medications, and having visited the emergency department due to HTN, did not show a significant effect after adjustment to the regression model (p > 0.05).

**Table 5:** Multivariate regression analysis to determine the significant independent factors associated with patients’ knowledge of their blood pressure readings (n = 465) ^§^ P-value calculated using chi-square test. ** Significant at p < 0.05. AOR: adjusted odds ratio; CI: confidence interval.

Factor	AOR	95% CI	P-value^§^
Age group			
≤55 years	Ref		
>55 years	0.839	0.466-1.510	0.558
Educational level			
High school or lower	Ref		
University degree or higher	3.027	1.569-5.839	0.001**
Time since diagnosis			
≥5 years	Ref		
<5 years	1.464	0.826-2.595	0.192
Regularly measure blood pressure			
Yes	2.913	1.652-5.136	<0.001**
No	Ref		
Compliance with blood pressure medication			
Yes	1.131	0.625-2.047	0.685
No	Ref		
Complications due to hypertension			
Yes	0.949	0.552-1.632	0.850
No	Ref		
Visited the emergency department due to high blood pressure			
Yes	1.096	0.635-1.890	0.742
No	Ref		
Rate your understanding of your chronic high blood pressure			
Good	Ref		
Acceptable	0.177	0.087-0.362	<0.001**
Weak	0.475	0.265-0.852	0.013**
Has a healthy lifestyle been discussed with you?			
Yes	3.368	1.999-5.674	<0.001**
No	Ref		

## Discussion

The present study was conducted to determine the knowledge of hypertensive patients regarding their BP readings and to identify the factors that influence this knowledge. Our results indicate that there is satisfactory knowledge among hypertensive patients regarding their target BP readings. Nearly three-quarters (74.4%) of our patients were aware of the importance of achieving their BP targets, and 77.8% of our patients had an acceptable to good understanding of chronic HTN. Several studies have reported that patients with HTN are aware of their disease, its significance, their target BP values, the associated risks, and treatment options [2-4]. Alshammari et al. [5] reported the highest awareness of BP readings at 85.1%, while Mirzaei et al. [6] reported the lowest awareness among patients about their disease at 49.7%. However, in a report by Sengul et al., they discovered an increasing trend of awareness about HTN diagnosis, from 40.7% in 2003 to 54.7% in 2012 [7]. A systematic review study done by Pereira et al. [8] showed BP control was significantly higher in older individuals than younger ones, and in women than men. Therefore, the knowledge of patients with HTN is increasing in all aspects over time; however, controlling their BP is challenging, especially among patients in low-quality HTN programs as these programs vary significantly between regions [9-11].

In this study, several factors were significantly associated with knowledge about BP readings, including age group, education level, time since diagnosis, regularly measuring BP, compliance with BP medications, complications due to HTN, visits to the emergency department due to HTN, level of understanding of chronic HTN, and having received education about healthy lifestyles. However, in our adjusted model, only education level, regularly measuring BP, level of understanding of chronic BP, and having received education about healthy lifestyles remained significant. Increased knowledge about BP readings was associated with a higher education level, measuring BP regularly, and receiving education about a healthy lifestyle; decreased knowledge of BP readings was associated with having an acceptable or poor understanding of chronic HTN. A large population-based cohort study done in China by Sun et al. [12] showed better BP control in individuals in middle school or above compared with individuals in elementary school or below.

However, these findings are inconsistent with those of a study by Mirzaei et al. [6]. In their adjusted model, increased awareness was positively associated with older age, female sex, and history of diabetes mellitus. Further investigation is required to identify the factors that influence patients’ knowledge of the different aspects of HTN.

The advantages of having a high level of education are supported in the literature. Patients with a higher level of education are more likely to have better perspectives regarding their disease. Bakhsh et al. reported that highly educated patients demonstrated better awareness and self-management practices regarding their disease than less educated patients [13]. A study conducted in the Makkah region revealed that knowledge about HTN, risk factors, and treatment was significantly associated with a high educational level but not with age or sex [14]. These findings are consistent with our study, as we observed a significant relationship between the knowledge of BP readings and education level, where a higher level of education was associated with a higher likelihood of having adequate knowledge of BP targets.

Most of our patients showed increased knowledge and good practices regarding their disease and its management. For example, 97.8% of the patients received medications for HTN, while compliance with HTN medications was 79.1%. Furthermore, 61.1% of patients had received education about the goal of BP measurement, and approximately 72.9% had received information about healthy lifestyles. However, the patients’ interest in enhancing their knowledge about some aspects of their disease was estimated to be lower. Only 43.4% of our patients showed interest in healthy food and sports, 27.5% showed interest in the complications of HTN, and only 22.2% showed interest in HTN treatments. In Iran, the use of prescribed anti-hypertensive medications was reported by 71.5% of patients; however, only 38.9% were able to control their BP [6]. Therefore, the author emphasized the importance of interventions for increased screening coverage. In Sri Lanka, most patients demonstrate good practices in measuring BP readings; 95% of patients regularly check their BP every 12 months, and 71% of patients have good compliance with anti-hypertensive medications [15]. Of the remaining patients who have poor compliance, the most reported reason is forgetfulness, which was also detected among our patients. In our study, 14.8% of respondents indicated forgetfulness as the most common reason for nonadherence to anti-hypertensive medications. The need for programs to improve the awareness of long-term complications of uncontrolled HTN and appropriate management to control BP is imperative, and it is essential to achieve better outcomes and quality of life among this population.

The preferred learning resource for HTN education among our patients was face-to-face discussion (50.5%); explanatory videos were the second most preferred option (32%), and written publications were the least preferred option (16.8%). The preferred source of HTN information was social media (39.1%) followed by social gatherings (28.4%) and the doctor (12.3%). These results contrasted those of Oliveria et al., who found that the most common source of information for hypertensive patients was a physician or other healthcare provider followed by mass media (59%) and friends and relatives (30%) [3]. However, in a study by Alharbi et al., the respondents complained that their doctors did not provide adequate information about HTN, and 89.2% of respondents claimed that their doctors underestimated informing them about the risks of anti-hypertensive therapy [14]. The role of healthcare providers, including doctors, is vital in the management and treatment of patients as they are the most reliable source of information, and appropriate information should be provided to patients to avoid mismanagement and potential complications of the disease. Therefore, reinforcing efforts to enhance prevention, early prognosis, and treatment of HTN is imperative.

## Conclusions

Hypertensive patients who visited the outpatient clinic at King Fahad Hospital had a good understanding of their target BP readings. Being educated, regularly measuring BP, and having received education about healthy lifestyles were associated with increased motivation to achieve BP targets. Awareness campaigns are needed to educate patients with HTN about the importance of controlled BP, and the role of healthcare providers is vital in this population. More research is needed to obtain more insight into the knowledge of hypertensive patients regarding how they manage their BP and to determine the factors that influence this knowledge.

## References

[1] (2022). Health threats from high blood pressure. https://www.heart.org/en/health-topics/high-blood-pressure/health-threats-from-high-blood-pressure.

[2] Yi-Bing W, De-Gui K, Long-Le M, Le-Xin W (2013). Patient related factors for optimal blood pressure control in patients with hypertension. Afr Health Sci.

[3] Oliveria SA, Chen RS, McCarthy BD, Davis CC, Hill MN (2005). Hypertension knowledge, awareness, and attitudes in a hypertensive population. J Gen Intern Med.

[4] Wolf-Maier K, Cooper RS, Banegas JR (2003). Hypertension prevalence and blood pressure levels in 6 European countries, Canada, and the United States. JAMA.

[5] Alshammari SA, Alajmi AN, Albarrak RA (2021). Quality of life and awareness of hypertension among hypertensive patients in Saudi Arabia. Cureus.

[6] Mirzaei M, Mirzaei M, Bagheri B, Dehghani A (2020). Awareness, treatment, and control of hypertension and related factors in adult Iranian population. BMC Public Health.

[7] Sengul S, Akpolat T, Erdem Y (2016). Changes in hypertension prevalence, awareness, treatment, and control rates in Turkey from 2003 to 2012. J Hypertens.

[8] Pereira M, Lunet N, Azevedo A, Barros H (2009). Differences in prevalence, awareness, treatment and control of hypertension between developing and developed countries. J Hypertens.

[9] NCD Risk Factor Collaboration (NCD-RisC) (2019). Long-term and recent trends in hypertension awareness, treatment, and control in 12 high-income countries: an analysis of 123 nationally representative surveys. Lancet.

[10] Saeed AA, Al-Hamdan NA, Bahnassy AA, Abdalla AM, Abbas MA, Abuzaid LZ (2011). Prevalence, awareness, treatment, and control of hypertension among Saudi Adult population: a national survey. Int J Hypertens.

[11] (2019). World Health Survey - Saudi Arabia 2019. https://www.moh.gov.sa/en/Ministry/Statistics/Population-Health-Indicators/Documents/World-Health-Survey-Saudi-Arabia.pdf.

[12] Sun K, Lin D, Li M (2022). Association of education levels with the risk of hypertension and hypertension control: a nationwide cohort study in Chinese adults. J Epidemiol Community Health.

[13] Bakhsh LA, Adas AA, Murad MA, Nourah RM, Hanbazazah SA, Aljahdali AA, Alshareef RJ (2017). Awareness and knowledge on hypertension and its self-care practices among hypertensive patients in Saudi Arabia. Ann Int Med Dent Res.

[14] Alharbi SA, Wedhaya MA, Alluqmani MF, Alrehaili SS (2017). Evaluation of knowledge in hypertensive Saudi population in Makkah, KSA. Egypt J Hosp Med.

[15] Ralapanawa U, Bopeththa K, Wickramasurendra N, Tennakoon S (2020). Hypertension knowledge, attitude, and practice in adult hypertensive patients at a tertiary care hospital in Sri Lanka. Int J Hypertens.

